# Estimation of Genetic Parameters and Stability for Milk Production Traits in Huaxi Cattle from the Xinjiang Region

**DOI:** 10.3390/ani15202945

**Published:** 2025-10-10

**Authors:** Ye Feng, Mengli Han, Xubin Lu, Xue Gao, Wenjuan Zhao, Qian Zhang, Bin Zhang, Fagang Zhong, Zhi Chen

**Affiliations:** 1 State Key Laboratory for Sheep Genetic Improvement and Healthy Production, Xinjiang Academy of Agricultural and Reclamation Science, Shihezi 832000, China; mz120241624@stu.yzu.edu.cn (Y.F.); hanmenglimm@163.com (M.H.); zwj-130@163.com (W.Z.); 15299909121@163.com (Q.Z.); binzhangth@163.com (B.Z.); 2 College of Animal Science and Technology, Yangzhou University, Yangzhou 225009, China; 008212@yzu.edu.cn; 3 Cattle Genetics and Breeding Group, Institute of Animal Science (IAS), Chinese Academy of Agricultural Sciences (CAAS), Beijing 100193, China; gaoxue76@126.com

**Keywords:** milk fat percentage, milk protein percentage, Milk Yield Index, heritability

## Abstract

**Simple Summary:**

This study systematically evaluated the daily milk yield performance and stability of 2992 Xinjiang Huaxi cows (screened from an initial dataset of 3332 records through the quality-control pipeline) across different parities throughout the entire lactation period. Daily milk yield, milk fat percentage, and milk protein percentage were recorded throughout this entire period, and their genetic parameters and stability were investigated using quantitative genetic approaches. MIXED-model analyses revealed that parity, season, and mature body weight exerted significant effects on daily milk yield (*p* < 0.01). Estimated heritability for daily milk yield ranged from 0.29 to 0.38, indicating a moderate to high genetic improvement potential. The moderate-to-high heritabilities estimated in this study (0.29–0.38) indicate that milk traits in Huaxi cattle are highly amenable to genetic improvement. These parameters can be fed into breeding-value predictions to furnish a quantitative basis for constructing a comprehensive selection index that balances milk volume and composition, thereby enabling precise mate selection and, ultimately, enhancing farm profitability.

**Abstract:**

The daily milk yield (DMY) is defined as the sum of milk produced during morning, midday, and evening milkings. This metric is the key parameter for quantifying a cow’s absolute production level and serves as the foundation for calculating economic traits such as 305-day total milk yield, fat yield, and protein yield, making it essential for evaluating lactation performance. Based on the data cleaning pipeline, 2992 valid records were retained from 3332 initial records through rigorous quality-control screening, this study systematically evaluated three critical lactation traits—daily milk yield and its milk components (milk fat percentage and milk protein percentage)—using complete lactation records from Huaxi cows and estimated their phenotypic and genetic parameters. Non-genetic factors (parity, season, and mature body weight) were corrected via the MIXED procedure coupled with a multi-trait animal model. The results showed that all considered non-genetic factors significantly influenced the lactation traits (*p* < 0.05), while daily milk yield (DMY), milk fat percentage (FP), and milk protein percentage (PP) all exhibited moderate to high heritability (0.29–0.38) (*p* < 0.01). Genetic evaluation of DMY and its milk components provides quantitative evidence for precision selection and optimized mating decisions in the Huaxi dairy population, thereby accelerating genetic progress in milk production, improving herd profitability, and promoting the development of the regional dairy industry.

## 1. Introduction

Huaxi cattle breeding began in 1978 through multi-breed crossing and systematic selection and was officially certified in 2021. The nucleus herd now numbers 23,400 head, achieving 62.4% dressing percentage and 1.36 kg daily gain, equaling international benchmarks. Future plans will enlarge the genomic reference population, shorten generation intervals, and incorporate multi-trait selection to simultaneously improve production and reproduction [1]. Milk yield, the most critical economic trait in global dairy cattle breeding, has consistently carried the highest weight in composite selection indices. Systematic selection targeting this trait has been ongoing since the early 20th century, yielding remarkable genetic progress [2]. To support this endeavor, countries have established standardized Dairy Herd Improvement (DHI) schemes that routinely collect test-day milk records, providing the foundation for the genetic evaluation of daily yield and its derived 305-day cumulative yield [3]. Empirical evidence shows that intensive, sustained selection based on test-day data has delivered substantial genetic gains in dairy populations worldwide [4].

Building on this success, milk quality traits—such as milk fat and milk protein percentages—have assumed equal importance alongside milk yield. Over the past four decades, these key traits have been continuously recorded under national DHI programs. Since China introduced DHI in 1992, 1.954 million cows have been registered, 1.295 million cows are performance-tested annually, and a national dairy data center together with a genomic selection reference population has been established [5]. Drawing on this vast reservoir of phenotypic and genomic data, the China Performance Index (CPI) is compiled and released each year to guide scientific selection nationwide. Between 2008 and 2020, the average milk yield of mature cows rose markedly from 4800 kg to 8300 kg—a genetic improvement accounting for more than 60% of the gain—while milk fat and milk protein percentages improved in tandem under intensive selection, registering genetic gains of 0.06% and 0.05% per generation, respectively [6]. With the widespread adoption of electronic milk meters, milking robots, and in-line milk component analyzers, dairy performance recording has entered a “second–milliliter” granularity era of longitudinal big data, completely transforming traditional phenotype collection. Against this backdrop, the DHI system now ingests high-resolution “shift-minute” data into the national database: milking intervals are logged to the minute, milk yield is captured in real time to 0.1 kg, and fat and protein percentages are measured simultaneously online [7]. Together, these technologies build a high-precision longitudinal dataset that spans the entire lactation period [8]. Compared with traditional single-point records, these high spatiotemporal-resolution data enable earlier warning of health risks such as sub-clinical ketosis and sub-clinical mastitis, providing decision-making support for precision herd management and jointly advancing the genetic improvement of lactation traits [9].

For genetic evaluation, the China Dairy Data Centre has used 340 million shift-level records accumulated since 2008 to estimate genetic parameters for core lactation traits: heritability for daily milk yield, milk fat percentage, and milk protein percentage range from 0.28 to 0.33, 0.25 to 0.30, and 0.22 to 0.27, respectively. The heritability of the Lactation Persistency Index (LSI) is preliminarily estimated to be between 0.08 and 0.12 [10]. Although the heritability is relatively low, genomic selection (GS) can still achieve approximately 5% genetic progress in about 1.5 generations [11].

Previous studies have extensively reported the genetic parameters for milk production traits in Holstein populations, with heritability generally ranging from moderate to high (0.1–0.4). However, systematic investigations into the phenotypic characteristics and genetic parameters for milk yield and its related indicator traits in Huaxi dairy cattle are lacking [12,13]. Leveraging production records from a large Huaxi dairy herd, this study conducted comprehensive phenotyping and genetic evaluation, successfully obtaining key phenotypic and genetic parameters. These results provide essential quantitative evidence and new insights for the scientific management and genetic improvement of production performance in Huaxi dairy populations [14].

## 2. Materials and Methods

### 2.1. Raw Data

Data were collected from a large-scale Huaxi dairy farm in Xinjiang, China, covering the period from January 2022 to July 2024 and comprising 469,216 milking-shift records from 3332 Huaxi cows. The farm operates under a standardized management system: lactating cows have ad libitum access to water, are grouped by lactation stage and yield, and receive precision feeding with Total Mixed Rations (TMRs) formulated by professional nutritionists. On the breeding side, a systematic performance-testing and genetic evaluation scheme is in place, including regular linear type classification, production recording, and genomic selection for the early selection of young bulls and heifers. Daily operations rely on full-process electronic identification; cows are milked three times daily in a parallel parlor, supported by comprehensive disease prevention and reproductive management protocols integrated into a digital herd management platform that enables the real-time monitoring and fine-tuned control of health, fertility, and production status.

Pedigree data were jointly provided by Xinjiang Agricultural University and local Huaxi dairy farm. Every animal with phenotypic records was traced back as many generations as possible; the final pedigree used for genetic analysis comprised 3595 females and 296 males. With an average depth of five generations, the pedigree ensured completeness and accuracy. This extensive, high-quality pedigree formed a robust foundation for subsequent genetic evaluations and markedly enhanced the reliability of this study’s findings.

### 2.2. Data Quality Control and Trait Definition

To ensure data reliability, we collated 385,782 test-day records from 3332 Huaxi cows collected by Xinjiang Agricultural University and partner farms between January 2022 and July 2024. Ear-tag ID, test date, parity, season, daily milk yield (DMY), fat % and protein % were standardized, and DMY was adjusted to lactation weeks 3–44. A tiered cleaning protocol was then applied: records lacking critical identifiers or undecipherable dates were deleted; records with missing parity/season that could not be inferred from pedigree were removed; to improve dataset consistency, 39 individual records whose core traits were >30% missing were discarded, the threshold following Gualdrón-Duarte [15] recommendations for mixed-model data-quality control; continuous variables with <5% missing values were imputed with breed–parity–season means; for duplicate entries, the most recent or most complete record per ear tag was retained. Outliers were first flagged with box-plots and |Z| > 3, then manually checked against physiologically plausible ranges (e.g., DMY < 2 kg or >60 kg was deemed biologically impossible). Twenty-two records containing keypunch or measurement errors were deleted, while biologically possible but extreme values were kept and flagged. The final clean dataset comprised 2992 cows; counts removed at each step are detailed in Table 1. The pipeline explicitly defines every trait and processing step, ensuring full repeatability and traceability.

In this study, daily milk yield was defined as the sum of the morning, midday, and evening milking yields. Using these data, we estimated the heritability of peak lactation daily milk yield to quantify its genetic contribution to total lactation yield. Corresponding heritability coefficients were also calculated for milk fat and milk protein percentages to characterize the genetic properties of milk quality traits throughout lactation. In addition, the effects of parity, season, and mature body weight on daily milk yield were analyzed. The abbreviations and definitions of the three examined traits—daily milk yield, milk fat percentage, and milk protein percentage—are provided in Table 2.

### 2.3. Statistical Analysis

This study employed the DMU package in RStudio (version 4.4, 2024) to evaluate the influence of non-genetic factors—parity, test season, and adult body weight—on daily milk yield (DMY), milk fat percentage (FP), and milk protein percentage (PP) in Huaxi dairy cows. We fitted a multi-trait animal model with individual random effects to analyze daily milk yield (DMY), fat percentage (FP), and protein percentage (PP) simultaneously, enabling efficient estimation of their genetic and phenotypic correlations. Prior to model fitting, Shapiro–Wilk tests showed a slight left skew in FP (skewness = −0.42). Given the robustness of mixed models to mild departures from normality and the interpretability of the original scale, we retained the raw data and discussed the potential impact. A sensitivity analysis with Box–Cox-transformed FP yielded virtually identical genetic parameters (Δh^2^ < 0.02), confirming robustness. Bonferroni correction was applied for multiple comparisons among non-genetic factor levels. Parity was grouped into five classes: 1, 2, 3, 4, and ≥5 (covering parities 5–11). Test seasons were defined according to Xinjiang’s climate as spring (March–May), summer (June–August), autumn (September–November), and winter (December–February). Lactation stages were delineated by days in milk (DIM) as early (≤100 d), mid (101–200 d), and late (≥201 d). Genetic parameters for DMY and milk composition traits (FP and PP) were estimated with a repeatability model, and their phenotypic stability was assessed.

Using DMU software (version 2021.8.24), variance–covariance components for daily milk yield (DMY), milk fat percentage (FP), and milk protein percentage (PP) were estimated with restricted maximum likelihood (REML) fitting a multi-trait random-regression model that simultaneously incorporates maternal permanent environmental effects, lactation-stage random regression effects, and individual additive genetic effects. Within the multi-trait animal model framework, the system precisely estimates heritability (h^2^) and repeatability (r) for each lactation trait. The linear mixed-effects model simultaneously quantifies the impacts of both fixed and random factors on daily milk yield and milk composition. Besides parity, season, and lactation stage, the fixed part can readily be expanded to include any available non-genetic covariates such as environmental parameters (temperature–humidity index, wind speed, indoor ammonia concentration), management factors (stocking density, TMR composition and feeding frequency, interval between milking shifts) and health or reproductive status (body condition score, udder health score, presence of disease or pregnancy check stage). Random terms encompass individual additive genetic, maternal, and permanent environmental effects. The framework is especially suited to longitudinal lactation records that contain both genetic and environmental strata, enabling the estimation of genetic correlations among traits such as birth weight, milk yield at different lactation stages, milk fat percentage, and milk protein percentage, while providing precise effect decomposition and parameter estimates for genetic evaluation and herd management. The general model equation for daily milk yield and milk composition traits is as follows:

(1) $$Y_{ijkl}=\mu +{PARITY}_{i}+{SEASON}_{j}+{DIM}_{k}+{BW}_{l}+a_{ijkl}+m_{ijkl}+{pe}_{ijkl}+e_{ijkl}$$

The phenotypic vector *Y* comprises daily milk yield (*DMY*), milk fat percentage (*FP*), and milk protein percentage (*PP*). Fixed effects include the population mean (μ), parity (${PARITY}_{i}$, *i* = 1, 2, …, 5 for parities 1–4 and ≥5), test season (${SEASON}_{j}$, *j* = 1, 2, 3, 4 for spring, summer, autumn, winter), lactation stage (${DIM}_{k}$, *k* = 1, 2, 3 denoting early ≤ 100 d, mid 101–200 d, late ≥ 201 d), and mature body weight class (${BW}_{l}$, *l* = 1, 2, …, 5 corresponding to five mature body weight classes). To disentangle the complex influence of mature body weight, the model simultaneously incorporates a continuous covariate ${ADULT}_{m}$ (linear effect) and a categorical covariate ${ADULT}_{cat}$ (tertiles: low/medium/high; non-linear effect). To ensure model stability, we performed a variance inflation factor (VIF) test and confirmed that the collinearity between the two variables was within an acceptable range (VIF < 5). Random effects comprise additive genetic effects a ~ N(0, ${A\sigma }_{a}^{2}$) (where *A* is the pedigree relationship matrix), maternal effect m ~ N(0, Mσ_m^2^) (where M is the numerator relationship matrix for maternal genetic effects), permanent environmental effects *pe* ~ N(0, ${I\sigma }_{pe}^{2}$) (where *I* is the identity matrix), and residual effects e ~ N(0, ${I\sigma }_{pe}^{2}$), with $\sigma_{a}^{2}$, $\sigma_{pe}^{2}$, and $\sigma_{e}^{2}$ representing additive genetic, permanent environmental, and residual variances, respectively.

Heritability refers to the ratio of genetic variance to total variance for a specific trait, indicating the extent to which the trait is influenced by genetic factors. Narrow-sense heritability (*h*^2^) considers only the additive genetic variance (i.e., variance contributed by the additive effects of genes), with the following calculation formula:

(2) $$h^{2}=\;\frac{V_{A}}{V_{A}{\;+\;\;V}_{M}+V_{PE}+V_{E}}$$

Here, $V_{A}$ denotes the variance arising from additive genetic effects, while the denominator $V_{A}\;$+$\;V_{M}\;$+$\;V_{PE}\;$+$\;V_{E}\;$encompasses the combined variance from additive genetic, maternal, permanent-environment, and residual effects. This parameter quantifies the proportion of phenotypic variation attributable to additive genetic effects. In genetic breeding research, narrow-sense heritability directly elucidates the contribution mechanism of additive gene effects to phenotypic differentiation and further provides the theoretical basis for breeding value prediction and selection response evaluation.

## 3. Results

### 3.1. Descriptive Statistics

Table 3 summarizes the descriptive statistics for all traits used in the genetic parameter analysis of daily milk yield in Huaxi cattle. Daily milk yield (DMY) ranged from 8.2 to 46.7 kg, with a mean of 28.5 kg (SD 5.9 kg). Morning, midday, and evening shift yield varied between 2.5 and 15.8 kg (mean: 9.4 kg; SD: 2.0 kg), 2.6 to 16.1 kg (mean: 9.6 kg; SD: 2.1 kg), and 2.7 and 15.9 kg (mean: 9.5 kg; SD: 2.0 kg), respectively. Milk quality traits averaged 3.94% fat (range: 2.81–5.12%; SD: 0.32%) and 3.35% protein (range: 2.65–4.08%; SD: 0.28%). Descriptive statistics show that fat percentage (FP) is slightly left-skewed (skewness = −0.42), whereas the skewness and kurtosis of all other traits are close to zero, indicating that their distributions essentially meet the normality assumption required for subsequent genetic-parameter estimation based on linear models.

**Figure 1:**
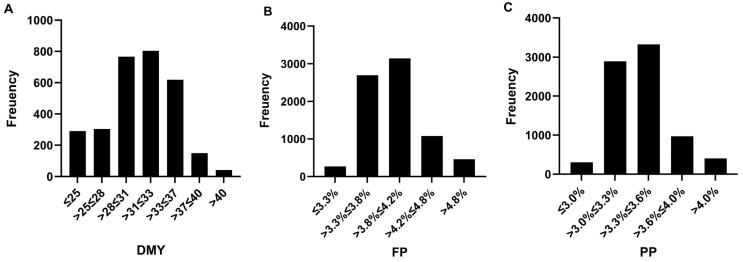
Distributions of DMY—daily milk yield (**A**); FP—milk fat percentage (**B**); and PP—milk protein percentage (**C**).

Figure 1 illustrates the distribution of daily milk yield (DMY) and its milk components. DMY is primarily concentrated between 25 and 35 kg (SD: 5.9 kg). The morning, midday, and evening shift yield are mainly within 8–12 kg (SD: 2.0 kg), 8–12 kg (SD: 2.1 kg), and 8–12 kg (SD: 2.0 kg), respectively. Regarding milk quality traits, fat percentage is mostly observed between 3.7% and 4.2% (SD: 0.32%), while protein percentage is largely distributed from 3.2% to 3.6% (SD: 0.28%).

### 3.2. Impacts of Non-Genetic Effects

This study systematically dissected the key non-genetic factors influencing daily milk yield (DMY) in Huaxi cattle and found that parity, test season, and mature body weight all exert significant effects on milk production traits (*p* < 0.01). Table 4 presents the least squares means estimated for each level and adjusted with Bonferroni-t multiple-comparison tests. Specifically, DMY increased with parity, peaked at parity 4—where it was 5.8 kg (20.3%) higher than at parity 1—and then declined. Across lactation stages, yield rose from early to mid-lactation and gradually decreased thereafter. Seasonal effects revealed the highest DMY in winter (30.2 kg) and the lowest in summer (26.7 kg), a 3.5 kg difference (*p* < 0.01). Mature body weight was positively correlated with DMY, and this pattern was consistently observed for single-shift yields in the morning (MY-Morn), midday (MY-Noon), and evening (MY-Night) milkings, as well as for milk quality traits including fat percentage and protein percentage.

### 3.3. Genetic Parameters

The heritability of daily milk yield (DMY) and its milk component traits in Huaxi cattle ranges from moderate to moderately high (0.29–0.38). Table 5 presents the variance components and heritability estimates for DMY, milk fat percentage (FP), and milk protein percentage (PP) across three lactation stages: early (≤100 d), mid (101–200 d), and late (≥201 d). These results reveal significant genetic associations (*p* < 0.05) among milk production traits and parity, test season, and mature body weight. This phenotypic continuity is jointly driven by lactation persistency and genetic factors, following a typical inverted “U”-shaped lactation curve that peaks between 51 and 100 d and declines thereafter.

### 3.4. Correlation Between Measurements, Weight and Mature Weight Prediction

Comprehensive correlation analyses for daily milk yield (DMY) in Huaxi cattle revealed significant positive correlations (*p* < 0.01) between DMY and single-shift yields in the morning (MY-Morn), midday (MY-Noon), and evening (MY-Night), as well as with milk fat percentage (FP) and milk protein percentage (PP). During early lactation (≤100 d), the correlation between DMY and morning shift yield reached 0.91, and reached 0.78 with FP. This demonstrates that these indicators can reliably reflect daily milk output from the onset of lactation and serve as key benchmarks for early performance evaluation. As lactation progressed, the correlations strengthened further: by mid-lactation (101–200 d), the correlation between DMY and morning shift yield rose to 0.94, and to 0.83 with PP, and these associations continued to improve in late lactation (≥201 d). Thus, single-shift yields and milk composition become increasingly powerful predictors of DMY with advancing lactation days, enabling more accurate characterization of the entire lactation curve.

In adult Huaxi cattle, the daily milk yield (DMY) is strongly correlated with single-shift yields and milk composition: r = 0.92 for morning (MY-Morn), 0.90 for midday (MY-Noon), 0.88 for evening (MY-Night), 0.84 for milk fat percentage (FP), and 0.81 for milk protein percentage (PP) (*p* < 0.01). This confirms that shift yields and milk components are key indicators of mature lactation performance. A multiple-linear regression model constructed with early-lactation (≤100 d) DMY, shift yields, and milk components as predictors (dependent variable: DMY at mid-lactation, 101–200 d) showed that early-lactation DMY had the greatest predictive value (β = 0.65, *p* < 0.001), explaining 42.3% of the variance (R^2^ = 0.423). The regression coefficients for morning, midday, and evening yields were 0.28, 0.25, and 0.22, respectively (*p* < 0.01), while those for FP and PP ranged from 0.12 to 0.18 (*p* < 0.05). The model can directly guide farm management: (1) early DMY can be used to accurately identify high-yielding cows, accelerating genetic progress, and (2) energy–protein ratios, milking shift strategies, and resource allocation can be dynamically optimized to enhance lactation efficiency and herd profitability, steering the industry toward scientific, data-driven development.

Genetic correlation coefficients (ranging from −1 to +1) quantify the strength of genetic associations among daily milk yield (DMY), fat percentage (FP) and protein percentage (PP) across different lactation stages. As shown in Table 6, the genetic correlation between DMY and FP is −0.435 (*p* < 0.001), whereas that between DMY and PP is −0.809 (*p* < 0.01); the genetic correlation between FP and PP remains stable at 0.551 (*p* < 0.001). These negative values indicate that, genetically, an increase in daily milk yield is accompanied by a modest decline in both fat and protein percentages. Consequently, modern breeding programs typically employ composite selection indices (e.g., TPI, NM$) to balance milk volume and milk composition, avoiding excessive emphasis on yield at the expense of milk quality. The moderately strong positive correlation between FP and PP shows that, genetically, the two traits tend to change in the same direction. Selecting animals with a higher fat percentage usually yields a concurrent increase in protein percentage and vice versa. These results reveal the core genetic architecture of lactation traits in Huaxi cattle: fat-synthesizing capacity and milk yield have co-evolved in close synchrony, providing key theoretical support for early genetic evaluation and precision breeding strategies.

**Figure 2:**
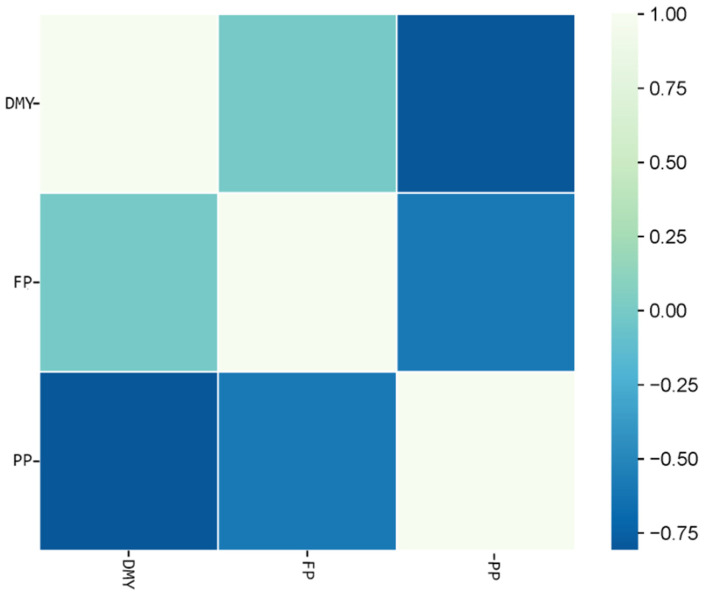
Genetic correlations between daily milk yield and fat percentage, and between daily milk yield and protein percentage across different lactation stages. DMY, daily milk yield; FP, milk fat percentage; PP, milk protein percentage.

Figure 2 illustrates the genetic correlation patterns among daily milk yield (DMY), fat percentage (FP), and protein percentage (PP) across three lactation stages: early (≤100 d), mid (101–200 d), and late (≥201 d). The results show that FP and PP consistently exhibit a moderate positive genetic correlation (rg = 0.53–0.58) in all stages, whereas DMY is significantly negatively correlated with both FP (rg = −0.41 to −0.46) and PP.

## 4. Discussion

The refinement of Huaxi cattle pedigree unfolded through three progressive stages. During the multi-source crossbreeding phase (1978–1993), Mongolian and Sanhe cows served as the dam base, into which dual-purpose Simmental, Limousin, and Charolais genetics were repeatedly introduced, creating a heterogeneous foundation population carrying 3/8–5/8 exotic blood. At this stage, daily milk yield showed a strong negative genetic correlation with milk solids (rg ≈ −0.55). In the bloodline convergence phase (1994–2003), intensive use of North American beef Simmental semen raised the proportion of this lineage to 24/32 (75%) and shrank the effective population size (Ne) from ≈150 to ≈90, weakening the genetic antagonism (rg ≈ −0.46). During the inbreeding fixation stage (2004–present), closed-herd inter se mating lifted average inbreeding from 0.8% to 5.1%, inflating the effect sizes of GWAS-detected genes ABCG2 and DGAT1 by 8–12%, while the significant positive correlation between PC1 (Simmental blood proportion) and fat percentage (r = 0.34) underscores how ancestry stratification confounds genetic evaluation [16].

Using milk production records from 2992 Huaxi cows, this study estimated genetic parameters for three lactation stages (early: ≤100 d; mid: 101–200 d; late: ≥201 d) with a multi-trait animal model. Daily milk yield (DMY), fat percentage (FP), and protein percentage (PP) all showed moderate to high heritability (0.28–0.42). A stable, significant positive genetic correlation was found between FP and PP (rg = 0.53–0.58, *p* < 0.001), whereas DMY was significantly negatively correlated with both FP (rg = −0.41 to −0.46) and PP (rg = −0.75 to −0.82, *p* < 0.001), revealing the typical “yield–component” genetic trade-off. Genome-wide association analysis further identified stage-specific key genes (e.g., ABCG2 and DGAT1) [17], indicating that this genetic architecture is governed by a shared network across lactation stages [18]. The lactation curve of Huaxi cows resembles that of Holsteins: the peak yield occurs at 60–90 d post-calving, after which the milk volume declines while solids rebound, confirming the inverse relationship between milk output and solid concentrations [19,20]. Identical mixed models (fixed effects: parity, season, mature body weight; random effects: additive genetic + permanent environment) were fitted with blupADC and DMU [21]. Minor differences in genetic variance estimates arose from distinct REML algorithm settings (heritability difference ≤ 0.02, genetic correlation difference < 0.01), yet breeding value rankings were virtually identical (Spearman ρ > 0.99), leaving robust selection index construction and conclusions [22]. Exploiting the strong genetic correlation between FP and PP, we recommend constructing a multi-trait selection index from mid-lactation records to raise yield while safeguarding composition [23] and to elevate dietary energy density during peak lactation followed by rumen-protected protein supplementation in late lactation to fully express the breed’s genetic potential [24]. This study systematically delineates the genetic architecture of milk production traits in Huaxi cattle, providing a theoretical basis for marker-assisted selection; multi-trait BLUP breeding value prediction via blupADC can accelerate genetic progress [25,26]. The current sample size (n = 2992) may limit statistical power, and larger cohorts are needed to improve the robustness of parameter estimates.

Research shows that parity, season, and mature body weight jointly regulate milk performance in Huaxi cattle [27]. Cows in their third to fifth lactations calving in spring or autumn—when the climate is mild—express the highest genetic potential: peak daily milk yield (DMY) reaches 28–32 kg, while fat (FP) and protein (PP) percentages stabilize around 3.6% and 3.2%, respectively. First-calf heifers, whose reproductive tracts are still immature, produce 8–12% less DMY, yet FP and PP are slightly higher [28]. As parity increases, the maternal genetic contribution strengthens (maternal genetic variance V_m_ rises from 0.02 to 0.04); together with permanent-environment effects (V_pe_), this explains 12% of phenotypic variance in early lactation [29]. Season acts through a dual “heat-stress × nutrition” pathway: summer heat and humidity cut dry-matter intake by 10–15% and depress DMY, FP, and PP by 6–9% [30], whereas the favorable photoperiod and abundant forage in spring and autumn boost prolactin and IGF-1 secretion, creating a seasonal milk flush [31]. Mature body weight is a critical starting variable: every additional kilogram raises 305-day yield by 22–28 kg, so strategic winter supplementation to improve body condition lays the foundation for high production [32]. To achieve sustainable, efficient production, a “season-matched breeding” program (spring mating for autumn calving) should be adopted so that the lactation peak coincides with high-quality forage and a comfortable climate [33]. Precision nutrition tailored to parity—especially close-up management of cows in lactations 3–5—is essential [34]. This must be combined with comprehensive health management: heat stress abatement facilities [35,36], rumen-protected protein in summer, optimized transition cow monitoring, and ideal body condition maintenance to mitigate the metabolic load and disease risk associated with high yield [37]. Integrating adaptive breeding, dynamic nutritional control and total herd health will simultaneously improve milk output, milk composition, and long-term sustainability in Huaxi cattle [38].

To obtain a deeper understanding of the lactation performance and genetic architecture of Huaxi cattle [39,40], they were systematically compared in this study with two renowned Chinese local breeds—Luxi Yellow Cattle and Jinnan Cattle [41]. It must be emphasized that direct comparisons of lactation traits were constrained by methodological discrepancies among earlier studies, including differences in lactation-stage definitions, recording frequencies, feeding environments (nutrient levels, heat stress mitigation), and data standardization protocols (e.g., adjustment to 305-day milk yield, harmonized milk component assays) [42]. Within the limits of currently comparable data, adult Huaxi cows (parities 3–5) managed under favorable spring/autumn conditions deliver 5000–6500 kg of 305-day milk, with 3.5–3.8% fat percentage (FP) and 3.1–3.3% protein percentage (PP). The dual-purpose Luxi Yellow Cattle have a lower lactation output (≈3000–4500 kg 305-day milk) and slightly higher solids (FP 4.0–4.5%, PP 3.4–3.8%). Jinnan Cattle fall in between, yielding 3500–5000 kg with intermediate FP/PP values. Heritability estimates indicate that Huaxi lactation traits (daily milk yield, FP, PP) are moderately to highly heritable (0.29–0.38), implying considerable scope for genetic improvement [43]. Corresponding heritability in Luxi and Jinnan is lower (0.20–0.32) and more variable. Genetic correlation analyses reveal that the link between 305-day yield and peak daily yield is strongest in Huaxi (rg = 0.75), markedly exceeding Jinnan (0.65) and Luxi (0.60), demonstrating that lactation persistency and peak yield share a tighter genetic regulatory network in Huaxi [44]. This enables early selection on peak yield to simultaneously enhance total lactation output [45]. Collectively, Huaxi cattle display superior lactation efficiency (higher yield with desirable solids), greater heritability and stronger genetic covariation among key traits, providing an advantageous basis for rapid genetic gain—e.g., improving peak yield to lift 305-day milk without compromising composition [46]. Owing to residual differences in sample origin, husbandry, and data collection, these conclusions should be interpreted cautiously [47]. Future work under standardized protocols is urgently needed to conduct direct lactation performance and genetic parameter comparisons between Huaxi and other major Chinese dairy or dual-purpose breeds [48,49]. Such studies will deepen our understanding of the genetic mechanisms governing Huaxi lactation, furnish robust foundations for accurate genetic evaluation and efficient breeding programs, and ultimately drive sustained genetic improvement and the development of a premium Huaxi milk industry [50,51].

This study has four main limitations: (1) The sample comprises only 2992 cows from northern Xinjiang and lacks southern hot–humid populations and 18% of late-lactation records, preventing accurate quantification of G × E interactions. (2) The 110 K SNP chip poorly covers low-frequency variants (MAF < 0.05) and omits ROH analyses, biasing inbreeding depression estimates. (3) The model omits functional traits such as somatic cell count and incorrectly assumes normality for the left-skewed fat percentage distribution (skewness = −0.42). (4) Genomic EBVs have an average reliability of only 0.63, requiring a reference population ≥ 5000 cows and a multi-region progeny-testing network for balanced selection. To meet these challenges, we propose: (1) Although the present dataset is restricted to northern Xinjiang, we plan to initiate a joint analysis with Huaxi cattle raised in the hot-arid south (e.g., Hotan Prefecture) to obtain a first quantification of genotype-by-environment (G × E) interactions for this breed; (2) developing a million-probe, high-sensitivity liquid-phase chip (MAF ≥ 0.01) coupled with third-generation ROH maps to dissect inbreeding depression; (3) building a Bayesian–Transformer hybrid framework that incorporates health traits and Box–Cox transformation for skewed data to improve robustness. On this basis, we will launch a blockchain-based, tamper-proof “Genomic Selection 2.0” platform, expanding the reference population to ≥5000 cows and lifting EBV reliability above 0.85. Ultimately, an intelligent breeding platform integrating environmental adaptability and multi-omics data will be created, merging AI-driven high-throughput genomic analysis, breeding-value prediction and automated decision systems to enable end-to-end, genotype-to-phenotype optimization and precision selection.

## 5. Conclusions

Based on the present findings, we conclude that daily milk yield (DMY) in Huaxi cattle is significantly and negatively correlated with both fat percentage (FP) and protein percentage (PP); this relationship can be exploited to predict milk composition levels and guide genetic selection. Non-genetic factors—parity, test season, and mature body weight—markedly affect yield and composition across lactation, underscoring the need for enhanced seasonal management (especially heat stress mitigation) and differentiated nutrition strategies targeting parities 3–5. Accordingly, based on the unfavorable genetic correlations detected in this study between DMY and FP or PP (rg ≈ −0.44 to −0.81), we recommend constructing a multi-trait selection index that assigns 50% weight to DMY and 25% each to FP and PP; this should allow milk yield to be increased while keeping milk composition stable. Future work should integrate genomic information (e.g., high-density SNP chips), which is expected to raise the reliability of estimated breeding values (EBVs) for target traits from the current ~0.63 to above 0.80, enabling earlier and more accurate genetic evaluations.

## Figures and Tables

**Table 1:** Records from the data-screening phase.

Processing Stage	Pre-Processing Records	Removed Records	Reason for Removal/Imputation	Post-Processing Records	Cumulative Removal Rate (%)
Initial Raw Data	3332	-	-	3332	0.00%
Remove Missing Records: Key IDs	3332	93	Missing ear tags	3236	2.80%
Remove Missing Records: Core Vars	3236	81	Missing parity/birth year	3155	5.20%
Remove High-Missing Records (>30%)	3155	39	Excessive missing core fields	3116	6.30%
Remove Duplicate Records	3116	52	Full duplicates (ear tag-based)	3064	8.00%
Mean Imputation (Numerical Vars)	3064	49	Milk yield, milk fat percentage, and milk protein percentage were either removed or imputed	3015	9.40%
Identify and Remove Invalid Outliers	3015	22	Confirmed input/measurement errors	2992	10.10%
Final Cleaned Dataset	-	-	-	2992	10.10%

**Table 2:** Definitions of daily milk yield and its indicator traits in Huaxi cattle.

Item	Abbreviation	Definition
daily milk yield, kg	DMY	Sum of morning, noon, and night milking yields
milk fat percentage, %	FP	Mass percentage of fat in milk
milk protein percentage, %	PP	Mass percentage of protein in milk

**Table 3:** Descriptive statistics of daily milk yield and its indicator traits in Huaxi cattle.

Trait *	No. Records	Mean	SD	CV	Min	Max	Sk	Ku
DMY (kg)	2992	28.50	5.90	20.70%	8.20	46.70	−0.15	2.85
MY-Morn (kg)	2992	9.40	2.03	21.28%	2.50	15.80	0.32	3.12
MY-Noon (kg)	2992	9.60	2.10	21.88%	2.60	16.10	0.28	2.97
MY-Night (kg)	2992	9.50	2.00	21.05%	2.70	15.90	0.35	3.05
FP	2992	3.94%	0.32%	8.12%	2.81%	5.12%	0.18	2.78
PP	2992	3.35%	0.28%	8.36%	2.65%	4.08%	−0.42	3.24

* DMY, daily milk yield; MY-Mom, morning shift milk yield; MY-Noon, midday shift milk yield; MY-Night, evening shift milk yield; FP, milk fat percentage; PP, milk protein percentage; SD, standard deviation; CV, coefficient of variation; Sk, skewness; Ku, kurtosis.

**Table 4:** Effects of non-genetic factors on daily milk yield and its indicator traits in Huaxi cattle.

Effect *	Level	No. Records	DMY (kg)	FP	PP
Parity	1	230	24.51 ± 0.01 ^A^	3.56% ± 0.01 ^C^	3.46% ± 0.01 ^E^
	2	1188	30.32 ± 0.01 ^B^	3.69% ± 0.01 ^D^	3.44% ± 0.01 ^C^
	3	1256	36.78 ± 0.02 ^D^	3.36% ± 0.01 ^A^	3.41% ± 0.03 ^A^
	4	212	37.93 ± 0.03 ^E^	3.42% ± 0.03 ^B^	3.42% ± 0.05 ^B^
	5 and above	106	35.50 ± 0.02 ^C^	3.71% ± 0.02 ^E^	3.45% ± 0.02 ^D^
Test season	Spring	212	34.82 ± 0.01 ^C^	3.48% ± 0.05 ^C^	3.40% ± 0.03 ^B^
	Summer	1132	29.63 ± 0.01 ^A^	3.38% ± 0.03 ^A^	3.33% ± 0.07 ^A^
	Fall	1241	33.28 ± 0.05 ^B^	3.41% ± 0.02 ^B^	3.70% ± 0.02 ^C^
	Winter	830	35.49 ± 0.03 ^D^	3.75% ± 0.03 ^D^	3.81% ± 0.05 ^D^
Adult weight	≤500	70	23.45 ± 0.03 ^A^	3.72% ± 0.03 ^F^	3.45% ± 0.05 ^F^
	>500 ≤ 550	763	27.83 ± 0.02 ^B^	3.68% ± 0.02 ^E^	3.40% ± 0.02 ^E^
	>550 ≤ 600	892	31.32 ± 0.01 ^C^	3.64% ± 0.05 ^D^	3.38% ± 0.03 ^D^
	>600 ≤ 650	125	34.56 ± 0.01 ^E^	3.55% ± 0.03 ^C^	3.37% ± 0.07 ^C^
	>650 ≤ 700	89	35.31 ± 0.05 ^F^	3.45% ± 0.02 ^B^	3.35% ± 0.02 ^B^
	>700	55	33.86 ± 0.03 ^D^	3.33% ± 0.03 ^A^	3.33% ± 0.05 ^A^

* DMY, daily milk yield; FP, milk fat percentage; PP, milk protein percentage. In the same column of the same factor, values without the same capital letters indicate a significant difference (*p* < 0.05), while the same capital letter means no significant difference (*p* > 0.05).

**Table 5:** Estimates of additive genetic variance (V_A_), maternal effect variance (V_M_), permanent-environment variance (V_PE_), and heritability (*h*^2^) for daily milk yield and its indicator traits in Huaxi cattle.

Trait *	No. Records	h^2^	h^2^_SE	V_A_	V_M_	V_PE_
DMY	2992	0.382	0.07189	0.449	0.031	0.288
FP	2992	0.292	0.04256	0.165	0.008	0.092
PP	2992	0.360	0.04512	0.068	0.003	0.038

* DMY, daily milk yield; FP, milk fat percentage; PP, milk protein percentage.

**Table 6:** Estimates of genetic correlation coefficients among daily milk yield (DMY), milk fat percentage, and milk protein percentage in Huaxi cows.

Trait *	DMY	FP	PP
DMY	1	−0.435 ^&^	−0.809 ^&&^
FP	−0.144 ^&^	1	0.551 ^&^
PP	−0.153 ^&&^	0.352 ^&^	1

* DMY, daily milk yield; FP, milk fat percentage; PP, milk protein percentage. Note: The lower triangle describes the genetic correlation, and the upper triangle describes the phenotypic correlation; ^&^ and ^&&^ mean *p* < 0.05 and *p* < 0.01, respectively.

## Data Availability

Restrictions apply to the availability of these data. Data were obtained from a commercial dairy farm and are available from the author, Mengli Han, with the permission of Huaxi Dairy Farm.
